# Measurement Indicators of Age-Friendly Communities: Findings From the AARP Age-Friendly Community Survey

**DOI:** 10.1093/geront/gnab055

**Published:** 2021-04-28

**Authors:** Kyeongmo Kim, Tommy Buckley, Denise Burnette, Seon Kim, Sunghwan Cho

**Affiliations:** School of Social Work, Virginia Commonwealth University, Richmond, Virginia, USA; School of Social Work, Virginia Commonwealth University, Richmond, Virginia, USA; School of Social Work, Virginia Commonwealth University, Richmond, Virginia, USA; School of Social Work, Virginia Commonwealth University, Richmond, Virginia, USA; School of Social Work, Virginia Commonwealth University, Richmond, Virginia, USA

**Keywords:** Age-friendly community, Environment, Evaluation, Person–environment fit

## Abstract

**Background and Objectives:**

Cities and counties worldwide have adopted the concept of “age-friendly communities.” These communities aspire to promote older adults’ well-being by providing a safe, affordable built environment and a social environment that encourages their participation. A major limitation in this field is the lack of valid and reliable measures that capture the complex dimensionality and dynamic nature of the aging–environment interface.

**Research Design and Methods:**

This study uses data from the AARP 2016 Age-Friendly Community Surveys (*N* = 3,652 adults aged 65 and older). The survey includes 62 indicators of age-friendliness, for example, outdoor spaces, transportation, housing, social participation, and community and health services. We randomly split the sample into 2 equal subsamples for confirmatory factor analysis (CFA) and structural equation modeling (SEM).

**Results:**

CFA results indicated that both the 5-factor model and the second-order factor model adequately fit the data. In the SEM 5-factor model, outdoor space (β = 0.134; *p* = .017), social participation (β = 0.307; *p* < .001), and community and health services (β = −0.149; *p* = .008) were associated with self-rated health, the outcome of interest. The path coefficients of housing and transportation were not significant. In the second-order factor model, people who lived in more age-friendly communities reported better self-rated health (β = 0.295; *p* < .001).

**Discussion and Implications:**

Our findings show that the Age-Friendly Community Survey measures demonstrate reliability and concurrent validity. To promote older adults’ well-being, practitioners, policymakers, and researchers should focus on improving their built and social environments. They can use these measures for short- and long-term planning, monitoring, and evaluating age-friendly community initiatives.

In 2020, the global population of persons aged 65 and older was estimated at 727 million. By 2050, their numbers are projected to exceed 1.5 billion and to represent 16.0% of the world’s population. In response to this challenging trend, cities and countries worldwide are adopting the concept of “age-friendly communities” (AFCs; Jeste et al., 2016; Lehning et al., 2017; Lui et al., 2009). As Greenfield et al. (2015) explain, AFC initiatives are designed to “engage stakeholders from multiple sectors within a typically local geographic area to make physical and/or social environments more conducive to older adults’ health, well-being and ability to age in place and in the community” (p. 191).

In 2005, the World Health Organization (WHO) introduced the concept of “Age-Friendly Cities” and launched a global project to assist 33 cities to incorporate issues of population aging into their urban planning (WHO, 2007). The WHO AFC framework comprises eight focal points: outdoor spaces and buildings, transportation, housing, social participation, respect and social inclusion, civic participation and employment, communication and information, and community support and health services (WHO, 2007). This initial project also included a comprehensive AFC guide and checklist to help city planners and policymakers incorporate these key features into current and future designs. In 2010, the WHO introduced the “WHO Global Network of Age-Friendly Cities and Communities” to strengthen partnerships, promote the exchange of best practices, and bolster evaluations of AFC initiatives (Beard & Montawi, 2015).

Cities and communities have since launched projects using the WHO AFC framework. In 2008, for example, Canada’s provincial government introduced the Manitoba AFC Initiative in 100 communities (Menec et al., 2014); and in 2017, New York was the first U.S. state to be designated age-friendly (WHO, n.d.). These and other age-friendly initiatives are expected to vary depending on context (Lui et al., 2009; Steels, 2015; Van Hoof et al., 2018). However, inconsistencies in program implementation and in establishing benchmarks for measuring outcomes create within- and across-site challenges for process and outcome evaluation (Greenfield et al., 2015; Kerr et al., 2012). The lack of valid and reliable measures that capture the complex dimensionality and dynamic nature of the aging–environment interface remains a major limitation in this field. More rigorous assessment methods and instruments are needed to monitor progress, refine and test theory, and effectively plan, implement, and evaluate the impact of AFC on individuals and communities (Torku et al., 2020).

The current study uses AARP survey data for older Americans to assess a promising measurement tool for use with AFCs. We begin with a brief overview of theoretical literature on person–environment (P–E) fit and then review important efforts on AFC measurement to date.

## Person–Environment Fit

Human ecology theory provides a useful framework for examining how individual-level physical, psychological, and social characteristics interact with features of the environment over time and how these interactions operate dynamically with larger systems to affect well-being. Lawton and Nahemow’s (1973) ecological theory of aging launched a widely used and still productive line of inquiry on how older adults continually adapt themselves and their environments to achieve a workable match between competence and environmental press. This match, which is referred to as the “zone of maximum performance and comfort,” is core to the P–E fit model (Lawton, 1990).

Research on AFCs has used the P–E model as an organizing framework since the early 2000s. Its heuristic value for extending and refining the model conceptually and empirically has helped to ensure its wide and enduring appeal. For example, scholars have distinguished between subjective and objective dimensions of both older adults’ preferences and needs and of their environments (Edwards et al., 2006; Kahana et al., 2003). Others have introduced temporal and human development perspectives to account for within- and between-cohort differences in P–E fit at a single time point and longitudinally (Iwarsson, 2005; Thomése & van Groenou, 2006).

Wahl et al. (2012) considered historical and cohort-related changes that affect P–E fit, which led them to conceptualize the environment in terms of its influence on older adults’ sense of agency and belonging. Calling for greater specificity in the P–E model, they emphasized the need to determine how the goodness of fit affects subjective (e.g., life satisfaction) and objective (e.g., health and social) outcomes (Lien et al., 2015). Wahl et al. also urged increased rigor in assessing P–E fit, including the extent and quality of fit between older adults’ abilities and their level of comfort with meeting perceived and actual daily needs.

### AFC Measurement

To measure and evaluate the effectiveness of the AFC framework requires a valid and reliable means of measuring “age-friendliness” that draws on fundamental features of the WHO AFC framework. This approach will reflect the original intentions of the AFC framework and will allow for a more systematic examination of outcomes that are of greatest interest and salience to older adults (Steels, 2015). The WHO (2015) issued a guide with a framework and indicators to help communities “monitor and evaluate progress in improving age-friendliness” (p. 7).

However, efforts to assess AFCs vary considerably in terms of focus (e.g., individual vs. community), perspective (objective vs. subjective), and priorities (residents vs. experts and policymakers). Dellamora et al. (2015) reviewed assessment tools for baseline and follow-up measurement of age-friendliness. Noting the lack of empirical data on measuring AFCs, they concluded that the Community Assessment Survey for Older Adults (CASOA; Polco, 2020) was the most favorable existing measure as it includes all eight AFC domains and it had been used in previous research. The CASOA survey tool is commercially available, but the need to purchase it may diminish its accessibility and use.

Several other empirical studies have sought to develop a measure for AFCs. Menec and Nowicki (2014) created the “Age-Friendly Survey” (AFS) to assess how aspects of AFCs were associated with self-rated health in a sample of older adults in Manitoba, Canada. The AFS consisted of 54 items, covering seven domains that were similar to the eight from the WHO AFC framework. The Age-Friendly Environment Assessment Tool (AFEAT; Garner & Holland, 2020) is a 10-item measure designed to appraise individuals’ perceptions “of their home and local communities, the resources within the environment and how well suited it is to meet their daily needs” (p. 194). The AFEAT showed good reliability (α = 0.75) and was associated with a higher quality of life and lower levels of loneliness in a sample of older adults in the United Kingdom (Garner & Holland, 2020). Finally, the Age-Friendly Cities and Communities Questionnaire (AFCCQ; Dikken et al., 2020) is a 23-item measure that is based on the eight WHO domains. The authors reported good psychometric properties in a sample of older adults in the Netherlands. The AFS, AFEAT, and AFCCQ have yet to be tested or validated in other samples or geographic regions.

Other researchers have drawn on existing data sets to conceptualize AFC environments. Lehning et al. (2014) used exploratory factor analysis to determine “age-friendly characteristics” and to examine how these features are related to the self-rated health of older adults in Detroit. Choi (2020) used data from the AARP, which is based on the eight WHO domains, to explore the relationship of older adults’ perceptions of the availability and importance of age-friendly features of communities with self-rated health and functional limitations.

In addition to efforts to develop surveys and indicators of AFCs, others have created evaluation tools (similar to CASOA) to evaluate AFCs. Buckner et al. (2019) describe their development and piloting of an instrument to evaluate age-friendly aspects of policies and practices in Liverpool, United Kingdom. They note the tool’s potential for cross-cultural and cross-geographic applications. Case studies are often used in AFC research*—*Jackisch et al. (2015) present a review of 33 such studies. This approach addresses the need to contextualize the evaluation of “age-friendliness” in a particular city or community and can thus be useful for local practice and policy and as a descriptive tool. This advantage notwithstanding, the frequency of the case study approach reflects the shortage of rigorous empirical measures for use across settings. Most case studies also have limited scope and replicability and are difficult to use for evaluating outcomes (Steels, 2015). They also tend to rely mainly on expert and policymaker assessments rather than the experiences of residents, although Menec et al. (2016) found that assessments by government officials were congruent with the subjective views of community members.

Another approach to measuring AFC is the AARP “livability index,” an online tool that provides information about how livable a community or neighborhood is for older adults (AARP, 2018a). This index rates a neighborhood (identified by ZIP code and city/county) in terms of housing (affordability and accessibility), neighborhood (access to life and work), transportation (safe and convenient options), environment (clean air and water), health (prevention, access, and quality), engagement (civic and social engagement), and opportunity (inclusion and possibilities). Livability scores are derived from multiple sources of data and policy documents in these domains (AARP, 2018a). The index is comprehensive and can be used to guide local governments, community organizations, and researchers (Guzman & Harrell, 2015; Zhang et al., 2020). A potential drawback of the index is that it may not reflect residents’ views. Separate from the livability index, the AARP AFC Surveys used in this article collected data in U.S. metropolitan areas to assess perceptions of how age-friendly their community is, among other measures (AARP, 2018b). AARP State Research developed a community survey to capture age-friendliness based on the WHO AFC framework and the livability index (see AARP, 2018b and Choi, 2020 for more information). However, these measures have not been tested empirically.

Despite important empirical and conceptual advances on the assessment of AFCs, there is still no adequate measure for use across samples and locales. As more cities and towns adopt AFC frameworks to improve the well-being of older adults, there is an urgent need for valid and reliable measures that capture the complex dimensionality of the aging–environment interface. Accordingly, we aim to assess the factor structure of age-friendly indicators in the AARP AFC Surveys and evaluate the effects of these constructs on older adults’ self-rated health.

## Method

### Data Sources

We used data from the AARP 2016 AFC Surveys, which have two stated purposes: (a) to identify and prioritize main focus areas by conducting a community needs assessment and (b) to create a baseline for communities to help older adults age in place (AARP, 2018b). AARP researchers selected the following 15 U.S. metropolitan areas on the basis of size and demographic composition: Alexandria, VA; Augusta, ME; Chula Vista, CA; DeSoto County, MS; Fort Collins & Loveland, CO; Grand Rapids, MI; Larimer County, CO; Maple Grove, MN; Mecklenburg County, NC; Monroe County, NY; New Orleans Parish, LA; Pittsburgh–Allegheny County, PA; Sioux Falls, SD; St. Petersburg, FL; and Warren County, KY. Using the methodology of the American Association for Public Opinion Research, they conducted 30-min telephone or in-person interviews with older adults in Summer, 2016 (*N* = 7,001). The average rates of cooperation, refusal, and response were 48%, 15%, and 6%, respectively. The AARP webpage provides details on methodology by area (AARP, 2016; Choi, 2020). The current study was approved by the Institutional Review Board of Virginia Commonwealth University.

### Sample

Our analyses are based on the 3,652 adults aged 65 and older. Table 1 presents the selected characteristics of the sample. About 60% were female, and about half were aged 65–74 years. The majority of respondents were non-Hispanic White (83%), followed by non-Hispanic Black (11%), Hispanic (2.5%), and other racial/ethnic groups.

**Table 1.** Demographic Characteristics for Random Split-Half Samples (*N* = 3,652)

	Total (*N* = 3,652)		Sample 1 (*n* = 1,826)		Sample 2 (*n* = 1,826)			
Characteristics	*n*	%	*n*	%	*n*	%	χ ^2^	*p*
Age (years)							3.0	.56
65–70	1,242	34	600	32.9	642	35.2		
71–74	613	16.8	307	16.8	306	16.8		
75–80	820	22.5	422	23.1	398	21.8		
81–84	351	9.6	176	9.6	175	9.6		
85–89	339	9.3	180	9.9	159	8.7		
90+	287	7.9	141	7.7	146	8.0		
Sex							1.83	.18
Male	1,442	39.5	741	40.6	701	38.4		
Female	2,210	60.5	1,085	59.4	1,125	61.6		
Race/ethnicity							7.63	.47
White	3,033	83.1	1,516	83.0	1,517	83.1		
Black	399	10.9	200	11.0	199	10.9		
Asian	14	0.4	4	0.2	10	0.5		
Hispanic	89	2.4	43	2.4	46	2.5		
Native American or Alaskan Native	25	0.7	12	0.7	13	0.7		
Native Hawaiian or Pacific Islander	3	0.1	2	0.1	1	0.1		
Other races	13	0.4	6	0.3	7	0.4		
Do not know	4	0.1	4	0.2	46	2.5		
Refused	72	2	39	2.1	33	1.8		

### Measures

#### Age-friendly environments

The AARP AFC survey adapted 62 indicators of age-friendly environments from the WHO Age-Friendly Cities initiative (WHO, 2007). Based on our literature review and WHO guidelines, we categorized age-friendly indicators as outdoor space and building, transportation, housing, health and community services, social participation, civic engagement, and community information. We hypothesized that the dimensions of respect and inclusion would be embedded in all domains as a basic principle of age-friendly environments.

Table 2 provides detailed information on the survey questionnaire. The items included all domains of age-friendly environments, for example, “Home modification and repair contractors who are trustworthy, do quality work, and are affordable,” “Sidewalks that are in good condition, safe for pedestrians, and accessible for wheelchairs,” and “A range of volunteer activities to choose from.” Respondents rated each dimension on a 6-point scale (0 = *does not exist*, 1 = *poor*, 2 = *fair*, 3 = *good*, 4 = *very good*, 5 = *excellent*). Higher composite scores indicate greater age-friendliness.

**Table 2.** Standardized CFA Factor Loadings for Age-friendly Environments Items

	Five factor		Second-order five factor	
Survey items	Est	*SE*	Est	*SE*
Factor 1: Outdoor space and building				
1. Sidewalks that are in good condition, safe for pedestrians, and accessible for wheelchairs or other assistive mobility devices	0.64	0.02	0.64	0.02
2. Well-lit, accessible, safe streets and intersections for all users	0.72	0.01	0.72	0.01
3. Audio and visual pedestrian crossings	0.63	0.02	0.63	0.02
4. Separate pathways for bicyclists and pedestrians	0.62	0.02	0.62	0.02
5. Well-maintained streets	0.62	0.02	0.62	0.02
6. Easy to read traffic signs	0.66	0.02	0.66	0.02
7. Enforced speed limits	0.61	0.02	0.61	0.02
8. Well-maintained parks with enough benches	0.79	0.01	0.79	0.01
9. Safe parks	0.77	0.01	0.77	0.01
10. Public buildings and spaces including restrooms that are accessible to people of different physical abilities	0.74	0.01	0.74	0.01
11. Conveniently located emergency care centers	0.75	0.01	0.75	0.01
12. Well-maintained hospitals and health care facilities	0.76	0.02	0.76	0.02
13. Neighborhood watch programs	0.62	0.02	0.62	0.02
14. Conveniently located public parking lots and areas to park including handicapped parking	0.75	0.01	0.75	0.01
15. Handicapped parking	0.65	0.02	0.65	0.02
Factor 2: Transportation				
16. Accessible and convenient public transportation	0.9	0.01	0.9	0.01
17. Affordable public transportation	0.89	0.01	0.89	0.01
18. Well-maintained public transportation vehicles	0.89	0.01	0.89	0.01
19. Timely public transportation	0.89	0.01	0.89	0.01
20. Safe public transportation stops or areas that are accessible to people of varying physical abilities	0.91	0.01	0.91	0.01
21. Special transportation services for people with disabilities and older adults	0.94	0.01	0.94	0.01
Factor 3: Housing				
22. Home modification and repair contractors who are trustworthy, do quality work, and are affordable	0.69	0.02	0.70	0.02
23. A home repair service for low-income and older adults which helps with things like roof or windows repairs	0.66	0.02	0.66	0.02
24. Seasonal services such as lawn work or snow removal for low-income and older adults	0.61	0.02	0.61	0.02
25. Well-maintained homes and properties	0.7	0.02	0.89	0.01
26. Affordable housing options for adults of varying income levels such as older active adults communities, assisted living and communities with shared facilities, and outdoor spaces	0.79	0.02	0.91	0.01
27. Homes that are built with things like a no-step entrance, wider doorways, grab bars in bathrooms, and first floor bedrooms and bathrooms	0.74	0.02	0.89	0.01
28. Well-maintained, safe low-income housing	0.68	0.02	0.68	0.02
Factor 4: Social participation				
29. Conveniently located entertainment venues	0.78	0.01	0.78	0.01
30. Activities geared specifically toward older adults	0.83	0.01	0.84	0.01
31. Activities that offer senior discounts	0.76	0.01	0.76	0.01
32. Activities that are affordable to all residents	0.79	0.01	0.79	0.01
33. Activities that involve both younger and older people	0.77	0.01	0.77	0.01
34. A variety of cultural activities for diverse populations	0.79	0.01	0.79	0.01
35. Local schools that involve older adults in events and activities	0.79	0.01	0.79	0.01
36. Continuing education classes or social clubs to pursue new interests, hobbies, or passions	0.82	0.01	0.82	0.01
37. Driver education or refresher courses	0.75	0.01	0.75	0.01
38. A range of volunteer activities to choose from	0.78	0.01	0.78	0.01
39. Volunteer training opportunities to help people perform better in their volunteer roles	0.81	0.01	0.81	0.01
40. Opportunities for older adults to participate in decision-making bodies such as community councils or committees	0.75	0.01	0.75	0.01
41. Easy to find information on available local volunteer opportunities	0.78	0.01	0.78	0.01
42. Transportation to and from volunteer activities for those who need it	0.75	0.01	0.75	0.01
43. A range of flexible job opportunities for older adults	0.82	0.01	0.82	0.01
44. Job training opportunities for older adults who want to learn new job skills within their job or get training in a different field of work	0.82	0.01	0.82	0.01
45. Jobs that are adapted to meet the needs of people with disabilities	0.8	0.01	0.8	0.01
46. Policies that ensure older adults can continue to have equal opportunity to work for as long as they want or need to regardless of their age	0.77	0.01	0.77	0.01
47. Access to community information in one central source	0.68	0.02	0.68	0.02
48. Clearly displayed printed community information with large lettering	0.69	0.02	0.69	0.02
49. Free access to computers and the Internet in public places such as the library, senior centers, or government buildings	0.7	0.01	0.7	0.01
50. Community information that is delivered in person to people who may have difficulty or may not be able to leave their home	0.74	0.02	0.74	0.02
51. Community information that is available in a number of different languages	0.71	0.01	0.71	0.01
Factor 5: Community and social services				
52. Affordable health and wellness programs and classes in areas such as nutrition, smoking cessation, and weight control	0.84	0.01	0.84	0.01
53. Affordable fitness activities specifically geared toward older adults	0.83	0.01	0.83	0.01
54. Conveniently located health and social services	0.82	0.01	0.82	0.01
55. A service that provides people to help seniors easily find and access health and supportive services	0.83	0.01	0.83	0.01
56. Affordable home care services including personal care and housekeeping	0.83	0.01	0.83	0.01
57. Easily understandable and helpful local hospital or clinic answering services	0.75	0.01	0.75	0.01
58. Well-trained certified home health care providers	0.77	0.01	0.77	0.01
59. Affordable home health care providers	0.77	0.02	0.77	0.02
60. A variety of health care professionals including specialists	0.74	0.02	0.74	0.02
61. Health care professionals who speak different languages	0.75	0.02	0.75	0.02
62. Respectful and helpful hospital and clinic staff	0.76	0.01	0.76	0.01
Outdoor spaces and buildings			0.84	0.01
Transportation			0.72	0.01
Housing			0.83	0.01
Social participation			0.87	0.01
Community and health services			0.91	0.01

*Notes:* CFA = confirmatory factory analysis; Est = estimate; *SE* = standard error. All factor loadings are significant at *p* < .001.

#### Self-rated health

Self-rated health was measured by the statement: “In general, would you say your health is …” (0 = *poor*, 1 = *fair*, 2 = *good*, 3 = *very good*, 4 = *excellent*). Higher scores indicate better self-rated health status.

### Data Analysis

We randomly split the sample into two subsamples for confirmatory factor analysis (CFA; *n* = 1,682) to test the factor structure of the age-friendly environment indicators and for structural equation modeling (SEM) to examine the association of the age-friendly environment dimensions and self-rated health (*n* = 1,682). The sample size was sufficient for CFA and SEM (Harrington, 2009; Kline, 2015), and the two subsamples had similar characteristics (i.e., age group, gender, race/ethnicity; Table 1). Using Mplus version 8.4 (Muthén & Muthén, 2019), we employed the full information maximum likelihood estimation method. This approach is among the best for handling missing data in CFA and SEM (Brown, 2015).

To begin, we conducted CFAs to test the factor structure of the 62 indicators of age-friendly environments. As all items were ordered categorically and consisted of six response options, we estimated CFA models using the WLSMV estimator (i.e., weighted least squares using a diagonal weight matrix with standard errors and mean- and variance-adjusted chi-square test statistic that use a full weight matrix; Muthén & Muthén, 2019). To determine whether the CFA models fit the data, we used the following fit indices: root mean square error of approximation (RMSEA) values close to 0.06 or below, comparative fit index (CFI), and Tucker–Lewis index (TLI) values close to 0.95 or greater (Brown, 2015; Kline, 2015). We also used standardized root mean square residual (SRMR) values less than 0.06 to determine a good fit. We examined problematic discriminant validity using the cutoff criterion, a factor correlation over 0.85 (Brown, 2015). We then conducted SEM to test whether the identified factor structure was associated with self-rated health. We used the same WLSMV estimator and fit indices that we used in the CFAs to evaluate the SEM models.

## Results

### Factor Structure of Age-friendly Indicators (CFAs With Sample 1)

To determine the factor structure of the age-friendly environment item, we estimated four CFA models: seven-factor, six-factor, five-factor, and second-order factor models. We respecified the models based on a lack of discriminant validity between factors. Based on previous literature and the research team’s assessment of content validity, we hypothesized and tested a seven-factor model: outdoor space and buildings, transportation, housing, health and community services, social participation, civic engagement, and community information.

Table 3 presents fit indices for the CFA models. Factor loadings for the seven-factor model ranged from 0.61 to 0.94 and all were significant (*p* < .001). Fit indices for the seven-factor model were good: χ ^2^(1,808) = 6,238.866, *p* < .001, RMSEA = 0.037 (90% confidence interval [CI] = 0.036–0.038), CFI = 0.960, TLI = 0.958, SRMR = 0.045. However, the factor correlation between social participation and civic engagement was 0.87, suggesting that these two factors might be measuring the same construct, indicating a lack of discriminant validity (Brown, 2015). We thus combined factors and respecified a more parsimonious model.

**Table 3.** Fit Indices for Confirmatory Factor Analysis Models

Fit index	Seven factor	Six factor	Five factor	Second-order factor
χ ^2^ (*df*)	6,238.866*** (1,808)	6,707.808*** (1,814)	6,776.974*** (1,819)	7,259.189*** (1,824)
RMSEA (90% CI)	0.037 (0.036–0.038)	0.038 (0.037–0.039)	0.039 (0.038–0.040)	0.040 (0.039–0.041)
CFI	0.960	0.956	0.955	0.951
TLI	0.958	0.954	0.954	0.949
SRMR	0.045	0.046	0.047	0.051

*Note:* RMSEA = root mean square error of approximation; CI = confidence interval; CFI = comparative fit index; TLI = Tucker–Lewis index; SRMR = standardized root mean square residual.

***p < .001.

We next estimated a six-factor model of age-friendly environment items. Factor loadings for the six-factor model ranged from 0.61 to 0.94 and all were significant (*p* < .001). Fit indices for the six-factor model were also good: χ ^2^(1814) = 6,707.808, *p* < .001, RMSEA = 0.038 (90% CI = 0.037–0.039), CFI = 0.956, TLI = 0.954, SRMR = 0.046. However, the factor correlation of social participation and community information was 0.86, suggesting that these two factors might be measuring the same construct and indicating a lack of discriminant validity (Brown, 2015). These findings suggested that we needed to treat social participation and community information items as a single measure.

We next examined a five-factor model of outdoor space and building, transportation, housing, health and community services, and social participation. Factor loadings for this model ranged from 0.61 to 0.94 and again, all were significant (*p* < .001). Fit indices for the five-factor model were also good: χ ^2^(1,819) = 6,776.974, *p* < .001, RMSEA = 0.039 (90% CI = 0.038–0.040), CFI = 0.955, TLI = 0.954, SRMR = 0.047. But the significant WLSMV chi-square test may be a function of sample size (Kline, 2015). Factor correlations between domains ranged from 0.58 to 0.82, demonstrating discriminant validity (Brown, 2015). Although transportation and outdoor spaces and buildings were highly correlated, transportation emphasized affordable and accessible transportation systems, while outdoor spaces and buildings included built environments such as parks, public buildings, and pedestrian safety (WHO, 2007). Model fit indices were similar for the seven-, six-, and five-factor models, and we favored the more parsimonious five-factor model (Kline, 2015). All five factors showed good internal reliability: outdoor spaces and buildings (α = 0.91), transportation (α = 0.96), housing (α = 0.82), health and community services (α = 0.95), and social participation (α = 0.97).

We also examined a second-order factor model to see whether the first-order factors (i.e., outdoor space and building, transportation, housing, health and community services, and social participation) form a cohesive single scale for measuring age-friendly environments. Factor loadings ranged from 0.61 to 0.94 and all were significant (*p* < .001). Also, factor loadings for age-friendliness ranged from 0.72 to 0.91 and all were significant (*p* < .001). Similar to the five-factor model, fit indices for the second-order factor model were good: χ ^2^(1,824) = 7,259.189, *p* < .001, RMSEA = 0.040 (90% CI = 0.039–0.041), CFI = 0.951, TLI = 0.949, SRMR = 0.051. Again, the significant WLSMV chi-square test may be a function of sample size (Kline, 2015).

### SEM With Sample 2 (Concurrent Validity for Self-Rated Health)

We tested both the five-factor and the second-order factor SEM models to predict self-rated health because the two models equivalently fit the data.

#### The five-factor SEM model

**Figure 1.**
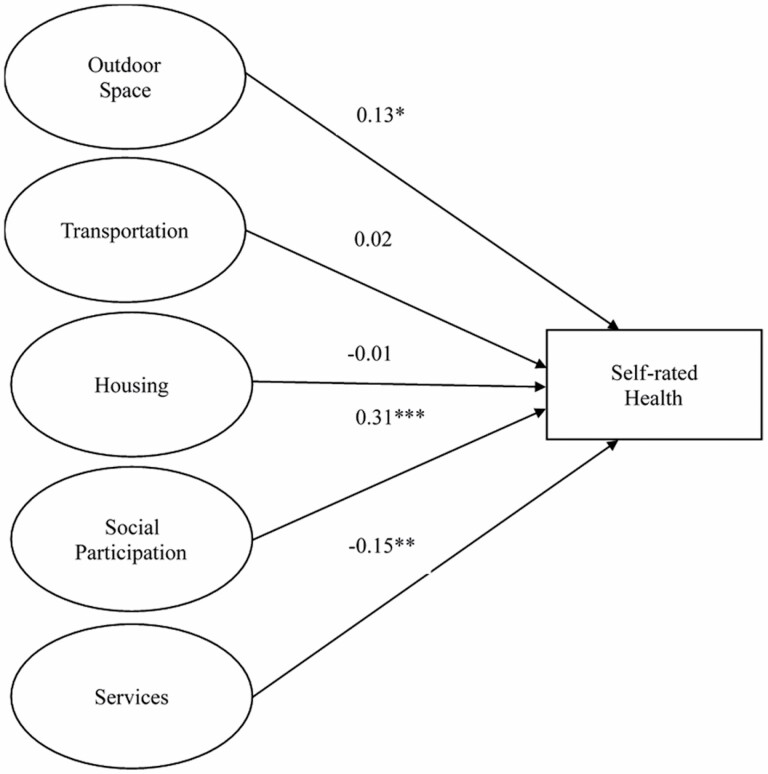
The five-factor structural equation model for self-rated health.

The five-factor SEM model yielded a good fit with the exception of a significant chi-square test (χ ^2^(1,876) = 6,848.867, *p* < .001); RMSEA = 0.038 (90% CI = 0.037–0.039), CFI = 0.956, TLI = 0.954, SRMR = 0.047. Figure 1 shows the five-factor SEM model predicting self-rated health. Standardized coefficients of outdoor spaces and buildings (β = 0.134; *p* = .017), social participation (β = 0.307; *p* < .001), and community and health services (β = −0.149; *p* = .008) were significantly related to self-rated health, even after adjusting for other factors; path coefficients of housing and transportation were not significant.

#### The second-order factor SEM model

**Figure 2.**
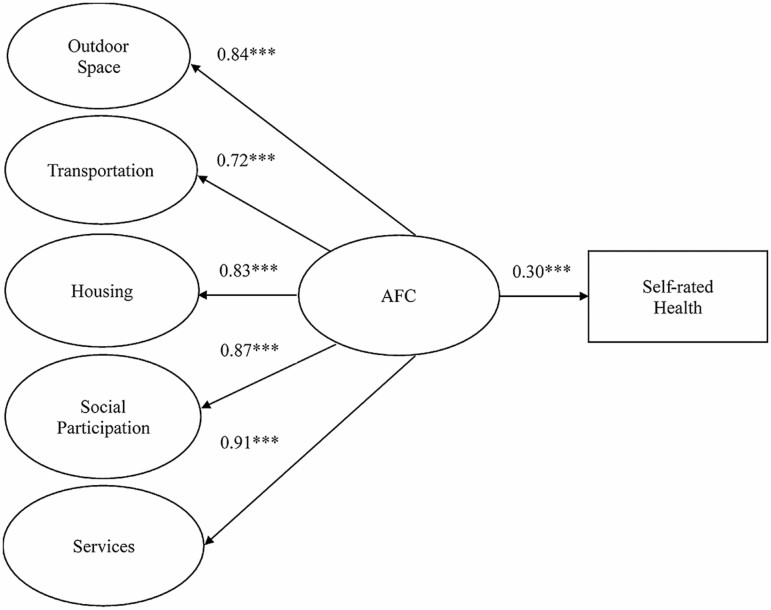
The second-order factor SEM model for age-friendly environments and self-rated health. AFC = age-friendly community; SEM = structural equation modeling.

The second-order factor SEM model also yielded an acceptable fit, but the chi-square test was significant (χ ^2^(1,885) = 7,303.505, *p* < .001): RMSEA = 0.040 (90% CI = 0.039–0.041), CFI = 0.952, TLI = 0.950, SRMR = 0.051. Figure 2 shows the second-order factor SEM model predicting self-rated health. Factor loadings ranged from 0.71 to 0.93 and all were significant. Standardized coefficients of age-friendliness (β = 0.295; *p* < .001) were significantly related to self-rated health, indicating that older adults who live in more AFCs reported better self-rated health.

## Discussion

This study is among the first to assess the reliability and validity of AARP AFC Survey measures. Our findings show that age-friendly indicators can be a useful tool for evaluating the age-friendliness of a community for older adults. CFA results indicate that both the five-factor model and the second-order factor model provide a good fit for the current older adult samples. Findings from the SEM models also suggest that both the five-factor model and the second-order factor models are predictive of older adults’ self-rated health.

Of the CFA models examined, social participation, civic engagement, and community information factors were highly related (correlations >0.85), which suggests low discriminant validity (Brown, 2015). The three factors measure very similar concepts and can be combined as a single measure. Consistent with previous literature, social participation is a broad concept that encompasses social activities and civic engagement (e.g., volunteering; Aroogh & Shahboulaghi, 2020; Douglas et al., 2017). Moreover, the concepts of community information and social participation may operate together, such that older adults may consider accessing and utilizing community information as a part of social participation. In fact, communities strive to make information more accessible in order to promote and sustain residents’ social participation. Our second-order model suggests that outdoor space and building, housing, transportation, community and health services, and social participation measure five distinct concepts within the larger AFC construct.

The goal of age-friendly initiatives is to create more favorable social and physical environments that will lead to improved health and well-being among older adults (Greenfield et al., 2015). Accordingly, we examined whether age-friendly environments predict older adults’ self-rated health. Previous studies report that older adults who live in communities that have accessible outdoor spaces and buildings have better physical health (Choi, 2020; Lehning et al., 2014). More accessible physical environments can lead to better health by promoting physical activities. Likewise, health-related benefits of social participation are well documented. Providing more opportunities can increase social interaction and potentially improve the health of older adults.

Our finding that higher levels of perceived availability of community and health services were associated with worse health outcomes was unexpected. Community health services potentially contribute to the well-being of all older adults, but those who need more community health services may be more likely to perceive the availability of the services. Other factors, such as transportation and housing, were not associated with self-rated health. While they are important factors in older adults’ quality of life, they may not be directly related to self-rated health. For example, transportation can help older adults participate in social activities and use of health services (Levasseur et al., 2017; Novek & Menek, 2014), but more evidence for a direct relationship is needed. One possible reason that housing is not associated with health (Burgard et al., 2012; Levasseur et al., 2017) is that affordable and available housing may affect one’s life satisfaction, but not directly affect self-rated health. Nonetheless, these findings confirm that, consistent with the goal of AFCs, improving both physical and social environments is essential to older adults’ health.

This study has several limitations. The data are from community-dwelling older adults in 15 metropolitan areas in the United States, so our findings cannot be generalized to older adults throughout the United States as well as across other demographics and countries. About 83% of the respondents were White. Disparities in access and quality of services exist across older racial and ethnic minorities (Dunlop et al., 2002; Hsieh & Ruther, 2017; Wilkins et al., 2020), and underlying constructs can differ among racial and ethnic groups. Future research should thus examine whether our CFA and SEM findings hold for diverse sectors of the older population.

The current coronavirus disease 2019 pandemic is a current example of disproportionately negative effects on the well-being of older adults of color, particularly those in low-income communities (Buffel et al., 2020). Researchers should determine whether and to what extent the concept of AFCs captures current and ongoing pandemic-related inequities in terms of social and spatial justice (Buffel et al., 2020; Martinez et al., 2020).

Adverse selection bias is another limitation of this study. Among older adults who have options, some select neighborhoods that are more likely to meet their needs. Healthy older adults are more likely to engage in social activities (Park, 2000) and they will presumably choose a neighborhood with more accessible outdoor spaces and more opportunities for social participation. Finally, this study used cross-sectional data, so we could not establish the predictive validity of the AFC factors. Future research should evaluate the effectiveness of an AFC using a treatment effect model or a longitudinal design.

Despite these limitations, our findings add to the growing literature on the measurement and effects of AFCs on older adult well-being. Our analyses confirm that age-friendly measures are a useful means to evaluate the age-friendliness of communities. More evidence to support construct validity is needed. Although self-rated health is a good measure when other health-related measures are not available (Lorem et al., 2020), future research should examine whether AFC factors have a stronger association with objective health and well-being outcomes, such as depressive symptoms, quality of life, or mortality. Also, AFC constructs need to be tested with different samples and methods over time. The underlying constructs of these measures, taken together and separately, also contribute to the advancement of theory. More rigorous assessment tools are needed to test and refine theory about the causes, correlates, and consequences of AFCs.

Reliable and valid measures of AFCs are indispensable tools for practitioners and policymakers who are responsible for planning, implementing, and evaluating AFCs at the local level. Likewise, researchers require such measures to develop and implement age-friendly programs and policy interventions and to evaluate their short- and long-term effects on older adults’ well-being. Using all 62 AFC indicators may not be feasible for practitioners and policymakers. Item reduction can be useful if the reduced items underscore and contribute to the construct (Boateng et al., 2018). Future research may reduce the number of indicators required to conceptualize AFC and evaluate a brief version of the AFC tool. The design and implementation of AFCs will of course be shaped by local context, but the basic components of all contexts will be interrelated and mutually reinforcing (Torku et al., 2020) and will be underpinned and informed by values of respect and inclusion. By advancing measurement in this field, this study adds to the rigor of research and to the ability to assess the effectiveness of AFCs for improving the well-being of community-dwelling older adults.
